# Supplementary material for: On the analysis of genetic association with long-read sequencing data
Source: PLoS Genet. 2025 Sep 29;21(9):e1011887. doi: 10.1371/journal.pgen.1011887 (PMC12500163; doi:10.1371/journal.pgen.1011887)
Supplement: S4 Text — (PDF) [file pgen.1011887.s004.pdf]

S4 Text: Relationships between RoP, saturated test and 4 df interaction test

Diplotypes	Additive		Dominance		Interaction and phase terms					Phasetypes			
	G_A	G_B	D_A	D_B	$G_A \cdot G_B$	$G_A \cdot D_B$	$D_A \cdot G_B$	$D_A \cdot D_B$	V	P_{cis}	D_{cis}	P_{trans}	D_{trans}
ab/ab	0	0	0	0	0	0	0	0	0	0	0	0	0
Ab/ab	1	0	1	0	0	0	0	0	0	0	0	1	1
aB/ab	0	1	0	1	0	0	0	0	0	0	1	0	0
AB/ab	1	1	1	1	1	1	1	1	1	1	1	0	1
Ab/aB	1	1	1	1	0	1	1	1	0	0	0	1	0
Ab/Ab	2	0	0	0	0	0	0	0	0	0	0	0	0
aB/aB	0	2	0	0	0	0	0	0	0	0	0	0	0
AB/aB	1	2	1	0	1	0	2	0	0	1	1	1	0
AB/Ab	2	1	0	1	1	2	0	0	0	1	0	1	1
AB/AB	2	2	0	0	4	0	0	0	0	2	0	2	0

Table 1: Genotypes, interaction terms, the phase term and the additive and dominance phasetypes of two biallelic loci with alleles A,a and B,b. The phasetypes P_{cis} and P_{trans} are coded with respect to allele A and B.

The additive *cis* and *trans* relationships can be written as the linear combinations of the phase term and the four genotype interaction terms as in Table 1:

$$P_{cis} = V + \frac{1}{2} \cdot (-G_A \cdot D_B + G_A \cdot G_B - D_A \cdot G_B + D_A \cdot D_B)$$

$$P_{trans} = -V + \frac{1}{2} \cdot (G_A \cdot D_B + G_A \cdot G_B + D_A \cdot G_B - D_A \cdot D_B)$$

Therefore, the saturated model, which tests the epistasis and phase effects by the 5 df test $H_0 : \beta_{G_A G_B} = \beta_{G_A D_B} = \beta_{D_A G_B} = \beta_{D_A D_B} = \beta_V = 0$, can fully capture the additive phase effects. However, the additional degrees of freedom reduce its power compared to the 1 df RoP tests.

The saturated test cannot effectively distinguish *cis* effects from *trans* effects. In particular, the direction of β_V relies on both *cis* and *trans* contributions as well as the reference alleles for the phasetypes. Below we summarize the directions of β_V:

Contributing phase relationships	Direction of β_V
Cis_{AB}	+
Cis_{Ab}	-
Cis_{aB}	-
Cis_{ab}	+
$Trans_{AB}$	-
$Trans_{Ab}$	+
$Trans_{aB}$	+
$Trans_{ab}$	-

The recessive inheritance patterns of *cis* and *trans* relationships are identical, i.e., $I_{cis=2} = I_{trans=2}$, where I is the indicator variable that equals 1 when $Cis_{AB} = 2$ or $Trans_{AB} = 2$, respectively. Both *cis* and *trans* relationships can represent recessive effects with their additive and dominance phasetypes:

$$I_{Cis=2} = \frac{1}{2}(Cis_{AB} - D_{cis}) = \frac{1}{2}(Trans_{AB} - D_{trans})$$

therefore, unlike the single locus analysis with genotypes, the power of RoP to detect recessive *cis* effects cannot be improved by simply including the dominance terms for *cis* and *trans* relationships and testing $H_0 : \beta_{P_{cis}} = \beta_{D_{cis}} = 0$, as the recessive *cis* effects can also well explained by the remaining additive and dominance terms of *trans* relationships.

Instead, the recessive *cis* effect can be modelled by the 4 interaction terms

$$I_{Cis=2} = \frac{1}{4} \cdot (-G_A \cdot D_B + G_A \cdot G_B - D_A \cdot G_B + D_A \cdot D_B)$$

and the 4 df interaction test has the best performance in detecting recessive *cis* effects, even though it doesn't include phase information.
