# Supplementary figures and images for: On the analysis of genetic association with long-read sequencing data
Source: PLoS Genet. 2025 Sep 29;21(9):e1011887. doi: 10.1371/journal.pgen.1011887 (PMC12500163; doi:10.1371/journal.pgen.1011887)

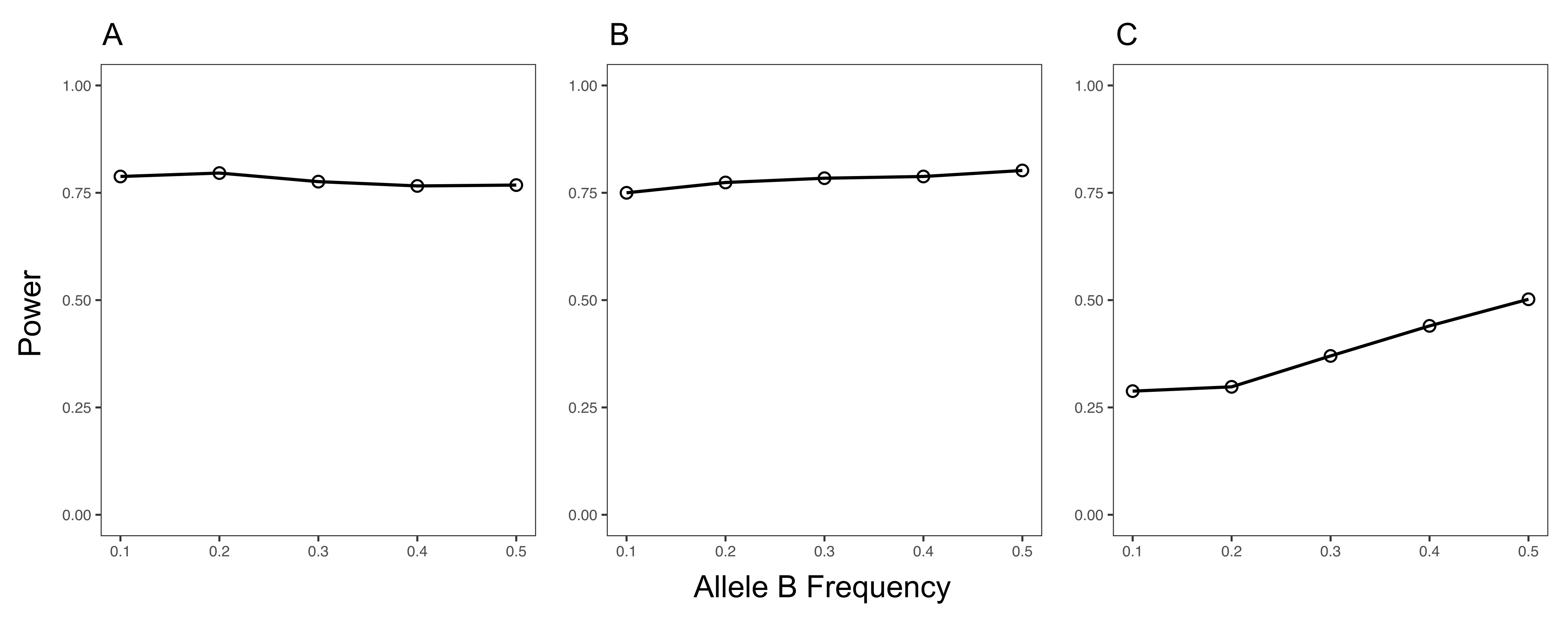

Supplement: S1 Fig — (A) Marginal effect contributed by allele A; (B) cis effects and (C) trans effects. In each scenario, haplotypes were simulated for n = 1,000 individuals, with PA=0.2 and PB ranging from 0.1 to 0.5, D’ = 0. A continuous phenotype was simulated by a linear model, with coefficients and residual standard deviation selected to maintain a heritability h2 = 0.01. The power of haplotype regression was then assessed by 500 iterations. (TIFF) [file pgen.1011887.s005.tif]

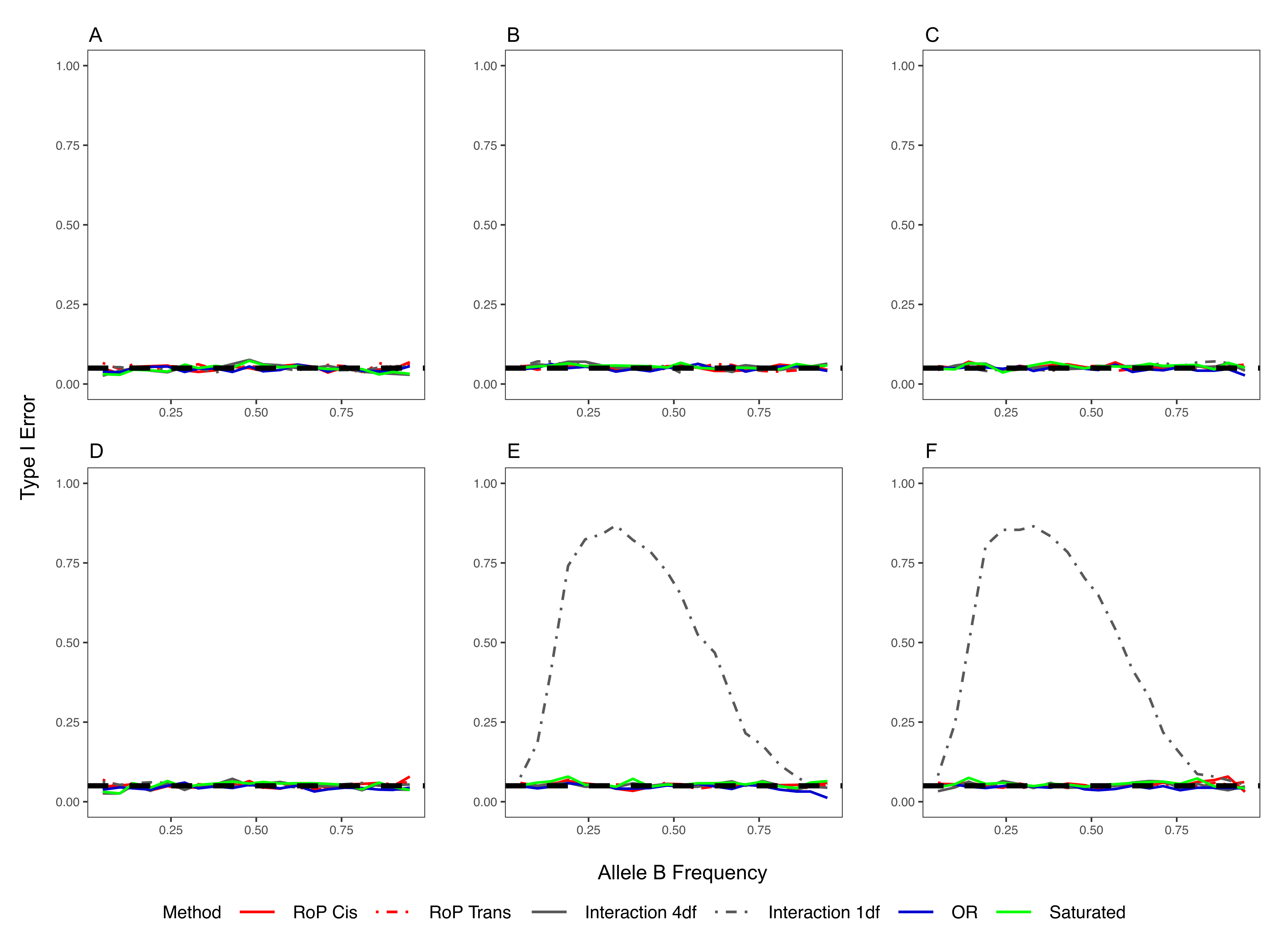

Supplement: S2 Fig — Additive (A)(D), dominant (B)(E) and recessive (C)(F) inheritance models were examined; (A)-(C): the variants are not in LD (D’ = 0) and (D)-(F): in LD (D’ = 0.8). Dashed lines correspond to a type I error of 0.05. (TIFF) [file pgen.1011887.s006.tif]

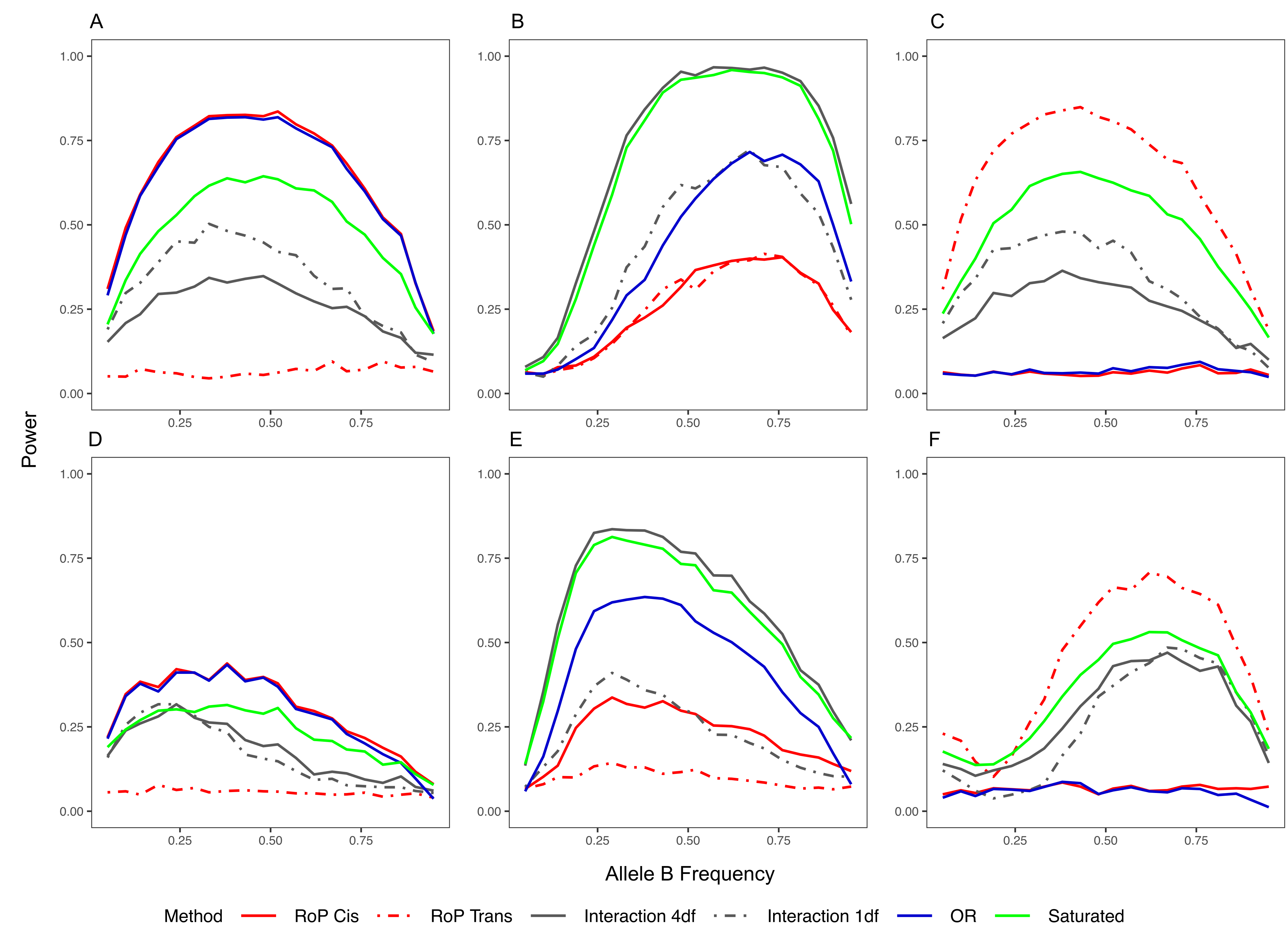

Supplement: S3 Fig — (A)(D) Dominant cis effects, (B)(E) recessive cis effect and (C)(F) dominant trans effects. (A)-(C): the variants are not in LD (D’ = 0) and (D)-(F): in LD (D’ = 0.8). (TIFF) [file pgen.1011887.s007.tif]

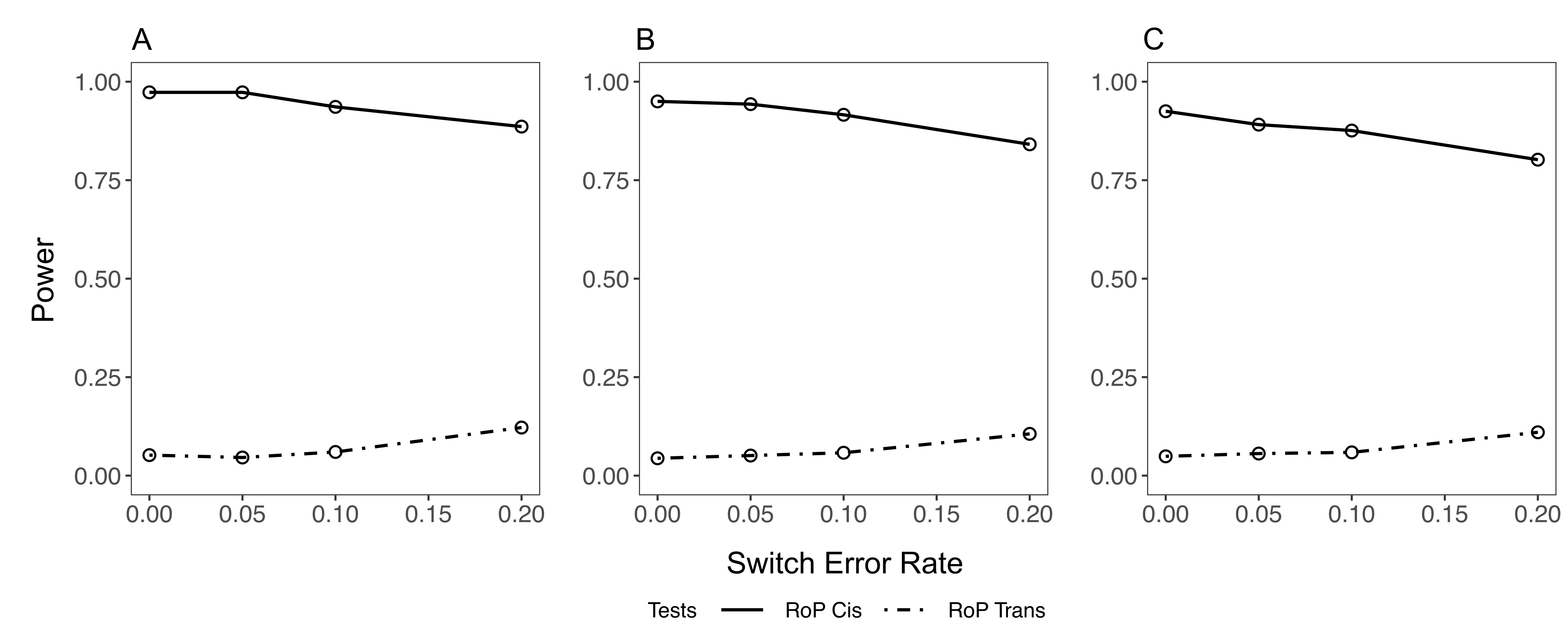

Supplement: S4 Fig — (A) Both alleles are rare with MAF = 0.05; (B) one rare allele (MAF = 0.05) and one common allele (MAF = 0.2); (C) both alleles are common with MAF = 0.2. For each scenario, haplotypes were simulated for n = 5,000 individuals. A continuous outcome was generated under an additive cis effect with heritability h2 = 0.003. Switch errors were introduced only in individuals heterozygous at both variants at rates of 5%–20%, with 0% (no switch error) as the benchmark. The power of detecting cis and T1E for trans effects at each error rate level were estimated from 2,000 iterations. (TIFF) [file pgen.1011887.s008.tif]
