# Supplementary material for: On the analysis of genetic association with long-read sequencing data
Source: PLoS Genet. 2025 Sep 29;21(9):e1011887. doi: 10.1371/journal.pgen.1011887 (PMC12500163; doi:10.1371/journal.pgen.1011887)
Supplement: S1 Table — “Yes” corresponds to the correct type I error rate. (PDF) [file pgen.1011887.s009.pdf]

S1 Table. Summary of simulation results for type I error. "Yes" corresponds to the correct type I error rate.

	RoP	Interaction 4 df and Saturated	Interaction 1 df	OR
D'=0				
Model 1: Additive effect on locus A	Yes	Yes	Yes	Yes
Model 2: Dominant effect on locus A	Yes	Yes	No	Yes
Model 3: Recessive effect on locus A	Yes	Yes	Yes	Yes
Model 4: Additive effects on both loci	Yes	Yes	Yes	No
Model 5: Dominant effects on both loci	Yes	Yes	Yes	No
Model 6: Recessive effects on both loci	Yes	Yes	Yes	No
D'=0.8				
Model 1: Additive effect on locus A	Yes	Yes	Yes	Yes
Model 2: Dominant effect on locus A	Yes	Yes	No	Yes
Model 3: Recessive effect on locus A	Yes	Yes	No	Yes
Model 4: Additive effects on both loci	Yes	Yes	Yes	No
Model 5: Dominant effects on both loci	Yes	Yes	No	No
Model 6: Recessive effects on both loci	Yes	Yes	No	No
