# Supplementary material for: On the analysis of genetic association with long-read sequencing data
Source: PLoS Genet. 2025 Sep 29;21(9):e1011887. doi: 10.1371/journal.pgen.1011887 (PMC12500163; doi:10.1371/journal.pgen.1011887)
Supplement: S2 Table — Switch errors were introduced only in individuals heterozygous at both variants at rates of 5%–20%, with 0% as the benchmark (no phasing error). Haplotypes were simulated for n = 5,000 individuals under an additive cis effect with heritability h2 = 0.003. Power for detecting cis effects and type I error (T1E) for trans effects were estimated from 2,000 iterations per error level under three MAF scenarios: Rare-Rare (MAF = 0.05 for both alleles), Rare-Common (MAF = 0.05 and 0.2), and Common-Common (MAF = 0.2 for both alleles). RoP retained over 95% of its original cis power and maintained nominal trans T1E up to 10% switch error, with substantial T1E inflation at 20%. (PDF) [file pgen.1011887.s010.pdf]

S2 Table. Impact of phasing uncertainty on RoP. Switch errors were introduced only in individuals heterozygous at both variants at rates of 5%–20%, with 0% as the benchmark (no phasing error). Haplotypes were simulated for n = 5,000 individuals under an additive *cis* effect with heritability $h^2 = 0.003$. Power for detecting *cis* effects and type I error (T1E) for *trans* effects were estimated from 2,000 iterations per error level under three MAF scenarios: Rare-Rare (MAF = 0.05 for both alleles), Rare-Common (MAF = 0.05 and 0.2), and Common-Common (MAF = 0.2 for both alleles). RoP retained over 95% of its original *cis* power and maintained nominal *trans* T1E up to 10% switch error, with substantial T1E inflation at 20%.

**Switch Error Rate**	**Rare–Rare**		**Rare–Common**		**Common–Common**	
	**Power (*cis*)**	**T1E (*trans*)**	**Power (*cis*)**	**T1E (*trans*)**	**Power (*cis*)**	**T1E (*trans*)**
0%	0.973	0.052	0.950	0.044	0.925	0.049
5%	0.973	0.046	0.943	0.051	0.891	0.056
10%	0.936	0.060	0.916	0.058	0.876	0.059
20%	0.886	0.122	0.841	0.106	0.802	0.110
