# Supplementary material for: On the analysis of genetic association with long-read sequencing data
Source: PLoS Genet. 2025 Sep 29;21(9):e1011887. doi: 10.1371/journal.pgen.1011887 (PMC12500163; doi:10.1371/journal.pgen.1011887)
Supplement: Attachment — Submitted filename: Response to Reviewers.docx [file pgen.1011887.s011.docx]

Dear Drs. Cordell, Zhu, Dudley and Goriely,

Thank you for the opportunity to revise and resubmit our manuscript “On the analysis of genetic association with long-read sequencing data” for consideration at *PLOS Genetics*. Please find below a detailed response to each of the three reviewers’ comments, which were comprehensive and extremely valuable. We revised our manuscript for resubmission accordingly, and for each response we also note the corresponding change in the revised manuscript where these recommendations have been incorporated.

Thank you again for considering our contribution and we hope with these changes you will find our manuscript suitable for publication in *PLOS Genetics*.

Sincerely,

Lisa J. Strug, PhD

Reviewer #1: A very readable paper.

The authors address an issue that is likely to be of increasing interest in particular in the context of understanding the role of enhancer and super enhancers and the modulation of pleotropic effects. The paper proposes and explores a model. Perhaps a few words on the implementation would be desirable. The comparison with existing approaches appears fair and the results plausibly explained. The authors focus on LRS.

Perhaps some comments on adapting the framework to accommodate phase uncertainty would be of interest, It could facilitate the use of short read sequences.

*Response: We thank the reviewer for the suggestion, which could enable the methodology to be used more broadly. So, although we conceptualized the development of the RoP for data with known, observed phase information, we have now evaluated RoP’s robustness to phasing error in short-read data in a new simulation study (pages 13 and 14, lines 432-450; Supporting Information S4 Fig and S2 Table). We simulated haplotypes for n=5,000 individuals with two independent variants under three minor allele frequency (MAF) scenarios: 1) both alleles are rare (MAF=0.05); 2) one rare allele and one common allele (MAF=0.05 and 0.2, respectively); and 3) both alleles are common (MAF=0.2). A continuous outcome Y was generated from their additive cis relationships, with effect size and residual variance chosen to yield a heritability of h^2^=0.003. Switch errors were introduced only in individuals who are heterozygous at both variants, at rates ranging from 5% to 20 %, with 0% (no switch error) serving as the benchmark. At a 10% switch error rate, RoP retained over 95% of its original power to detect the cis effect (relative to no switch error) and maintained nominal T1E for trans effects in all MAF scenarios, effectively distinguishing between the two mechanisms. Substantial inflation of T1E was observed at 20% switch error rate. Most modern phasing tools, such as SHAPEIT5, achieve switch error rates below 5% even for extremely rare variants (ref 42: Hofmeister et al., 2023), which is well below the error level at which RoP performance began to degrade in our simulations. These results demonstrate that under realistic short-read phasing accuracy with contemporary methods (ref 42: Hofmeister et al., 2023; ref 43: Browning et al., 2021), the statistical validity of RoP is preserved.*

Some typos could be corrected/clarified (e.g. page 31 after line 2 "++", or page 35 after line 1 "aa")

*Response: We thank the reviewer for the suggestion. Typos have now been corrected.*

**Reviewer #2:**

Summary

The authors propose a novel Regression on Phase (RoP) approach to detect phase effects of genetic variants on complex diseases, leveraging long-read sequencing (LRS) data. They demonstrate the method’s performance through simulation studies and application to cystic fibrosis (CF), revealing phase-dependent mechanisms that traditional GWAS approaches fail to capture. This is the first statistical method specifically developed to analyze both cis and trans phase effects in genetic association studies using LRS data. The methodology is well-presented, statistically sound, clearly explained, and supported by simulations and real data examples.

Comments

1. Theorem 1 (line 9): Should X_P be X_P^1 instead? Please clarify the notation.

*Response: We thank the reviewer for the comment. It should be X_P^1, and it is now corrected (page 5, line 124)*

2. The authors state that “The RoP test is designed to focus on testing the significance of the phase effects rather than the direction of the effects.” Can the authors elaborate on how the regression coefficients should be interpreted under this framework?

*Response: We thank the reviewer for the comment. When the reference alleles for the phasetypes are correctly specified, the phasetype coefficients provide unbiased estimates of phase effects, which is the expected change in phenotype per additional count of the phase relationship across the chromosome pair. However, when the reference alleles are misspecified, the effect size has the correct magnitude but may have an opposite direction relative to the true effect. For example, when the true risk phase relationship is* $Cis_{AB}$ *,* $P_{cis}=Cis_{ab}$ *yields the correct effect size, while the effect sizes for* $P_{cis}=Cis_{Ab}$ *and* $P_{cis}=Cis_{aB}$ *have the same magnitude as the true value but are opposite in sign.*

*Importantly, though, the predicted phenotype remains unchanged regardless of the choice of reference alleles. For example, a continuous outcome Y has the identical predicted values when P_cis_=Cis_AB_ and P_cis_=Cis_ab_. We provided the proof in S2 Text. We have now revised the corresponding paragraph in Methods (page 5, line 131-142) to make the interpretation clearer.*

3. Figure 2: Can the authors provide more explanation as to why RoP is sensitive to cis and trans phase effects but not to allelic heterogeneity? Additionally, why is T1E=0.05 included on the plot? It would be helpful if the authors also comment on the performance of the non-conditional method for comparison.

*Response: We thank the reviewer for the comment. RoP adjusts for the marginal effects while explicitly models the effects of the cis and trans configuration. As a result, it is sensitive only to the phase effects, not to allelic heterogeneity in which variants influence the phenotype independently.* *Other non-conditional approaches, such as epistasis tests, also control for marginal effects and detect the coordinated interaction. However, these methods cannot determine whether the association signal is driven by the cis or trans configuration. We added these clarifications in Results (page 8, lines 246-252). The T1E=0.05 was added as a reference to show RoP has nominal T1E under allelic heterogeneity. It is now clarified in the figure caption.*

4. Usability and scope: The method appears not suitable for large-scale or whole-genome analysis but rather for testing specific variants. Since exhaustive searches for epistasis signals across the genome are outside the intended scope, should multiple testing adjustments be considered when applying RoP? It would be useful if the authors could provide practical guidance on when and how to best apply the RoP test.

Furthermore, the absence of software implementation limits user accessibility. Can the authors clarify the recommended workflow? For example, should users first perform GWAS to identify variants of interest and then apply RoP? Are users expected to code genotype and phase data themselves? The development of a user-friendly tool would greatly facilitate adoption of this method.

*Response: We thank the reviewer for the suggestions. The RoP method has been implemented in the R package RegPhase, which is now available on GitHub (see Data and Code Availability). The current release includes the core functionality for the RoP test, supporting both biallelic and multi-allelic variants with integer allele coding. Future releases will add features that facilitate genome-wide applications, including VCF files input, LD block determination, haplotype merging, automatic allele coding, multiple testing correction.*

*While the RoP development and application in the manuscript mainly focused on analyzing GWAS significant loci, we now outline a framework for use that includes both GWAS significant loci and genome-wide analysis. A guideline was added to the Discussion section (pages 14 and 15, lines 489 - 511). For GWAS-significant loci with multiple association signals, RoP can be used to assess the phase effects between the lead SNPs of the primary and secondary signals, or between variants with potential functional relevance, as demonstrated through our SLC9A3 and SLC6A14 examples. In such targeted analysis, only one variant pair is tested for each locus, and multiple testing correction is not required. However, if multiple variant pairs are examined, appropriate multiple hypothesis testing adjustments should be applied.*

*For genome-wide analysis, we recommend first aggregating variants that are in strong LD into haplotypes and then testing phase effects between adjacent LD blocks using RoP, treating each LD block as a multi-allelic variant. The extension of RoP to accommodate multi-allelic variants is described in Method (pages 5 and 6, lines143 - 153). This approach avoids underpowered tests between highly correlated variants, reduces the multiple-testing burden, and improves efficiency. Further power gains can be achieved by merging similar haplotypes within each LD block (ref 17: Liu et al, 2008) prior to analysis, thereby reducing the number of alleles and lowering the degrees of freedom of the test. Because a single LD block can be paired with multiple others, the resulting RoP tests are correlated. Multiple-testing correction should therefore account for this dependency, for example, by using the effective-number-of-independent-tests approach (ref 51: Nyholt et al, 2004; ref 52: Li et al, 2005) or a permutation test rather than applying a conservative Bonferroni adjustment. Finally, methods developed for efficient epistasis screening (ref 23: Wan et al, 2010; ref 24: Crawfold et al, 2017; ref 49: Parée et al, 2010; ref 50: Soave et al, 2015) can be adapted to create a phase-aware RoP screening strategy that prioritizes variants for follow-up RoP testing. This framework is now provided in the revised manuscript Discussion.*

**Reviewer #3**:

The work introduced a novel regression on phase (RoP) method by encoding phase information into cis and trans. It can directly model and test the cis and trans effects of multiple genetic variants at a given genetic locus. Unlike traditional GWAS, which usually treat genetic variants as independent signals, RoP models can distinguish cis and trans phase effects between pairs of variants. Overall I find this manuscript well written with clearly presented results. I have a few specific comments:

1. The authors have provided examples of biallelic pairs, while in real world, multiple allelic variants may play a role together. The authors have stated that the RoP method can be applied in multi-allelic situation in the discussion section, but might lack a detailed description of how to extend into multi-allelic cases. It would be good if the authors can provide additional guidelines on how to further extend the method.

*Response: We thank the reviewer for the suggestion to include the multi-allelic RoP extension details, which are now included in Methods (page 5 and 6, line 143-153). Briefly, the extended model tests the phase effects between all non-reference alleles of each variant. For a variant A with m alleles (A1,A2,…,Am) and variant B with* n *alleles (B1, B2,…,Bn), the model includes additive genotypes (G) and the dominance terms (D) for non-reference alleles Ai and Bj (i,j >= 2), and the phase terms Cis(Ai Bj) and Trans(Ai Bj) for each non-reference allele pair. The cis and trans effects are tested across all allele pairs, each with (m-1)(n-1) degrees of freedom. Genotypes and phasetypes for the reference alleles (A1,B1) are omitted due to linear dependencies. This formulation allows RoP to handle LD blocks or other multi-allelic loci by treating each distinct haplotype within a block as an allele in the multi-allelic model, and handling LD blocks facilitates its application in genome-wide analysis.*

2. The authors applied the RoP methods to two loci and tested two non-phase and cis-phase cases. However, genome-wide analysis is missing, that is, whether the method can be computationally efficient and scalable. If the authors can wrap up the codes into a package, it would be beneficial to the community. At the same time, a description of suggested framework of how to apply RoP at genome-wide level would be helpful.

*Response: Yes, thank you, the RoP method is now implemented in the R package RegPhase which is available on GitHub; see Data and Code Availability. The current release includes the core functionality for the RoP test, supporting both biallelic and multi-allelic variants with integer allele coding. Future releases will add features that facilitates genome-wide applications, including VCF files input, LD block determination, haplotype merging, automatic allele coding, multiple testing correction.*

*We have now also included a framework for adapting RoP to genome-wide analysis in Discussion (page 14 and 15, 489-511). In brief, we recommend first aggregating variants that are in strong LD into haplotypes and then testing phase effects between adjacent LD blocks using RoP, treating each LD block as a multi-allelic variant. This approach avoids underpowered tests between highly correlated variants, reduces the multiple-testing burden, and improves efficiency. Further power gains can be achieved by merging similar haplotypes within each LD block (ref 17: Liu et al, 2008) prior to analysis, thereby reducing the number of alleles and lowering the degrees of freedom of the test. Because a single LD block can be paired with multiple others, the resulting RoP tests are correlated. Multiple-testing correction should therefore account for this dependency, for example, by using the effective-number-of-independent-tests approach (ref 49: Nyholt et al, 2004; ref 50: Li et al, 2005) or a permutation test rather than applying a conservative Bonferroni adjustment. Finally, methods developed for efficient epistasis screening (ref 23: Wan et al, 2010; ref 24: Crawfold et al, 2017; ref 49: Parée et al, 2010; ref 50: Soave et al, 2015) can be adapted to create a phase-aware RoP screening strategy that prioritizes variants for follow-up RoP testing. This framework is now provided in the revised manuscript Discussion.*

3. There have been haplotype regression methods available, which also include phase information to test the effects of each haplotype. Although the authors have included a haplotype OR test in simulations, to answer the question that whether RoP provides added value over simply modeling haplotypes categorically, it would be great to include a more explicit comparison to full haplotype regression and provide a comparison example on one real locus.

*Response: We thank the reviewer for the comment. Additional discussion on haplotype regression was added in the revised Method section (page 7, line 191-204).*

*In the biallelic case, haplotype regression is essentially equivalent to jointly testing the effect of the four cis relationships without adjusting for the marginal effects. Therefore, a significant result cannot indicate whether the signal was driven by independent main effects or from their coordinated phase configuration. Further, because only the cis relationships are modelled, haplotype regression dose not distinguish cis from trans effect, and it is under-powered in detecting trans effect. We include additional simulation results to support these arguments in Supporting Information S1 Fig.*

*RoP addresses these limitations by i) explicitly adjusting for marginal genotype effects and ii) providing separate, interpretable tests for cis and trans effects. As our simulation framework focused on phase-specific inference, we choose not to include haplotype regression as a benchmark in the current study.*

4. Phasing might be imperfect and misassigning haplotypes across variants can occur. If phasing errors occur, it can completely flip the assignment, turning cis into trans, and vice versa. It would be good to test the robustness in simulation studies by introducing different switch error rates.

*Response: We thank the reviewer for the comment which was also highlighted by Reviewer 1. Although RoP is primarily designed for long-read sequencing data where the phase information is observed, we have now evaluated RoPs robustness to phasing error in short-read data in a new simulation study (pages 13 and 14, lines 432-450; Supporting Information S4 Fig and S2 Table). We simulated haplotypes for n=5,000 individuals with two independent variants under three minor allele frequency (MAF) scenarios: 1) both alleles are rare (MAF=0.05); 2) one rare allele and one common allele (MAF=0.05 and 0.2, respectively); and 3) both alleles are common (MAF=0.2). A continuous outcome Y was generated from their additive cis relationships, with effect size and residual variance chosen to yield a heritability of h^2^=0.003. Switch errors were introduced only in individuals who are heterozygous at both variants, at rates ranging from 5% to 20 %, with 0% (no switch error) serving as the benchmark. At a 10% switch error rate, RoP retained over 95% of its original power to detect the cis effect (relative to no switch error) and maintained nominal T1E for trans effects in all MAF scenarios, effectively distinguishing between the two mechanisms. Substantial inflation of T1E was observed at 20% switch error rate. Most modern phasing tools, such as SHAPEIT5, achieve switch error rates below 5% even for extremely rare variants (ref 42: Hofmeister et al, 2023) which is well below the error level at which RoP performance began to degrade in our simulations. These results demonstrate that under realistic short-read phasing accuracy with contemporary methods (ref 42: Hofmeister et al, 2023; ref 43: Browning et al, 2021), the statistical validity of RoP is preserved.*

5. The effect size terms for cis and trans are novel and biologically meaningful. However, beyond their significance, the magnitude of these effect sizes also needs to be discussed. The authors might use several simulated or real examples to showcase what the predicted phenotype means for each phase type count, and compare the effect sizes with models that include haplotype effects or genotype effects only.

*Response: Thank you, this observation aligns with the request for clarity by Reviewer 2, Comment 2. Specifically, the predicted phenotype remains unchanged regardless of the choice of reference alleles. For example, a continuous outcome Y has the identical predicted values when P_cis_=Cis_AB_ and P_cis_=Cis_ab_. We have now provided a proof in S2 Text. When the reference alleles for the phasetypes are correctly specified, the phasetype coefficients provide unbiased estimates of phase effects; that is, the expected change in phenotype per additional count of the phase relationship across the chromosomes pair. However, when the reference alleles are misspecified, the effect sizes have the correct magnitude but may be opposite in direction relative to the true effect. For example, when the true risk phase relationship is* $Cis_{AB}$ *,* $P_{cis}=Cis_{ab}$ *yield correct effect sizes, while the effects sizes for* $P_{cis}=Cis_{Ab}$ *and* $P_{cis}=Cis_{aB}$ *has the same magnitude as the true value but opposite in sign. We revised the corresponding paragraph in the Method section (page 5, 131-142) to make this clear.*
